# Symbiotic simulation for the operational management of inpatient beds: model development and validation using Δ-method

**DOI:** 10.1007/s10729-019-09485-1

**Published:** 2019-06-03

**Authors:** David Oakley, Bhakti Stephan Onggo, Dave Worthington

**Affiliations:** 1 Department of Management Science, Lancaster University Management School, Lancaster University, Lancaster, LA1 4YX UK; 2 Southampton Business School, University of Southampton, Southampton, UK

**Keywords:** OR in health services, Symbiotic simulation, Validation, Bed management

## Abstract

In many modern hospitals, resources are shared between patients who require immediate care, and must be dealt with as they arrive (emergency patients), and those whose care requirements are partly known to the hospital some time in advance (elective patients). Catering for these two types of patients is a challenging short-term operational decision-making problem, since some portion of each resource must be set aside for emergency patients when planning for the number and type of elective patients to admit. This paper shows how symbiotic simulation can help hospitals with important short-term operational decision making. We demonstrate how a symbiotic simulation model can be developed from an existing simulation model by adding the ability to load the state of the physical system at run-time and by making use of conditional length-of-stay distributions. The model is parameterised using 18 months of patient administrative data from an Anonymised General Hospital. Further, we propose a new Δ-Method that is suitable for validating a stochastic symbiotic simulation model. We demonstrate the benefit of our symbiotic simulation by showing how it can be used as an early warning system, and how additional patient-level information which might only become available after admission, can affect the predicted bed census.

## Introduction

In many modern hospitals, resources such as beds, theatre time, medical equipment and staff are shared between patients who require immediate care, and must be dealt with as they arrive (emergency patients), and those whose care requirements are partially known to the hospital some time in advance (elective patients). Caring for these two types of patients poses a logistical challenge in the sense that some portion of each resource must be set aside for emergency patients when planning for the number and type of elective patients to admit. Hospitals have guidelines for the number of emergency patients they might expect to see in each planning period, although the exact number is unknown. If too many elective patients are admitted, the hospital’s ability to treat emergency patients will be reduced, potentially resulting in negative patient outcomes, such as having to turn patients away, and “outliers” - a term which refers to patients whose ward might not be ideally suited to their condition. On the other hand, if too few elective patients are admitted, patients can be left on waiting lists unnecessarily in the case of public health services, or hospital income can be lost in the case of private health care.

The potential benefits of using discrete event simulation (DES) models in health care are well established, and they are often preferred to other modelling approaches because of their ability to emulate the randomness seen in physical systems at a level of detail which is necessary for models to be convincing. Numerous literature surveys have tracked the progress of this modelling approach, including [1–5]. However, the use of DES is often limited to strategic or tactical decision making, and few have attempted to produce models which can help hospitals with short-term (operational) decision making. This is where symbiotic simulation can help.

Symbiotic simulation is a methodology in which there is a close relationship between a physical system and the simulation system that represents it. Based on the types of relationship between the physical system and simulation system, [6] classify symbiotic simulation into several categories. In this paper we describe research relevant to what they refer to as a *symbiotic simulation decision support system*. In this type of symbiotic simulation, the simulation reads data from the physical system regularly (i.e. to re-initialise the system state and if necessary, update the decision variables and/or simulation parameters). The simulation outputs are then used for what-if analysis, and an external decision maker can choose to change the behaviour of the physical system. In other words, the simulation system indirectly controls the physical system via the external decision maker, instead of an automatic actuator. As operational and real-time data becomes more readily available in health care, the use of symbiotic simulation in health care is becoming more feasible and some early work, for example [7, 8], is starting to appear. However, research into the application of symbiotic simulation in health care is still a long way behind industries such as manufacturing.

Our research aim for this paper is to investigate important issues associated with the development and use of *symbiotic simulation decision support systems* in the context of operational management of inpatients beds.

The challenges tackled in this paper are:

(i) symbiotic simulation model development – here we show how development can be achieved from an existing DES model by adding two functionalities: the ability to load the state of the physical system at run-time (to make predictions about how the physical system might evolve in the short-term) and the use of conditional length-of-stay distributions;
(ii) symbiotic simulation model validation – here we propose a new validation method, called ‘Δ-Method’;
(iii) symbiotic simulation model applications – here we demonstrate some of the benefits of a symbiotic simulation in a hospital context, in particular how it can be used as an early warning system, and how additional patient-level information (which might only become available after admission), can improve the accuracy of the simulation output.

In order to undertake this research we developed a whole-hospital, *proof-of-concept* symbiotic simulation model. We did this with the involvement of a real Anonymised General Hospital (AGH) for a period of about 18 months, after which we lost touch with them due to management changes. This relationship gave us exactly what was needed for this research. It provided us with a rich context, a full inpatient activity dataset for an 18 month period, and clear indications of how they would hope to use a symbiotic simulation, including the main performance measures that would interest them. Hence our *proof of concept* model is based on a conceptual model agreed with AGH staff, its validity is investigated by comparing model outputs versus actual performance, and its application is demonstrated based on realistic scenarios and real data sets.

The remainder of this paper is organised as follows. Section 2 provides an overview of traditional applications of simulation on hospital bed management, followed by a review of symbiotic simulation and its early applications in health care. In Section 3, we introduce the Anonymised General Hospital (AGH) and the traditional *proof-of-concept* DES model of the hospital that we developed with them. The main contributions of the paper are presented in Sections 4, 5 and 6. In Section 4, we show how a symbiotic simulation can be developed from an existing DES model, which may well reduce the development cost if such a DES model already exists, but also offers a viable approach even when starting from scratch. In section 5, we propose and demonstrate a new validation technique, called Δ-Method, which is suitable for validating symbiotic simulation models. In Section 6, we demonstrate how the symbiotic simulation can be used as an early warning system and how the additional information made available at simulation run-time can be used to improve the accuracy of the simulation output. Finally, we conclude the paper and highlight future work in Section 7.

## Literature review

Given the importance of achieving reasonable levels of efficiency in hospitals, bed management has been an active topic of research in Operational Research/Management Science (OR/MS) for a long time, resulting in numerous approaches to the problem and large quantities of related literature. The admission of elective patients can be viewed as a scheduling problem, and the literature in this domain is dominated by analytical methods which aim to provide optimal (or close to optimal) schedules given a set of constraints. With the proliferation of more powerful personal computers and programming languages, simulation has become one of the accepted tools in this domain that complement the analytical methods. The prevalence of simulation is due (in part) to its flexibility, which facilitates the modelling of complex systems, such as hospitals. In this section, we provide a broad review of the applications of simulation for bed management, followed by an introduction to symbiotic simulation and its applications including health care.

### Applications of simulation for bed management

Some of the earliest literature surveys in bed management include [9] whose surveyed papers investigate the relationship between admissions scheduling policies and hospital resources; [10] which focuses on the surgical scheduling literature, including “multiple constraint” models which account for bed numbers and nursing staff; and [11] whose survey covers the use of computer simulation across various healthcare systems, including admissions control and bed management.

Early work in this area includes [12], who developed a simulation model of a hospital treating narcotics addiction. While the type of patient differs significantly from the acute patients this research is concerned with, the model’s structure bears similarity to an acute hospital through the admission of non-authorized (unplanned) and authorized (planned) patients. The authors also note that the authorized patient stream is the most easily controlled, and therefore the decision variables for the model are based around their admission. The aim of the work is to minimise variation in the bed census while maintaining reasonable occupancy levels. Other early work includes [13], which uses a simulation model to test three routines for the development of an automated scheduler for elective admissions, with a focus on how estimating each patient’s length-of-stay might improve scheduling decisions.

[14] develops models for emergency admissions but they claim that it can be generalised to accommodate both emergency and elective streams. The work is interesting in that negative patient outcomes (crisis days and proportion of patients not admitted) are treated as a function of bed occupancy for a hypothetical acute English hospital. One of the most cited conclusions of the paper is that hospitals operating at 90% occupancy or higher will suffer regular crisis days, and that operating staffed but empty beds is a necessity for absorbing stochastic variation associated with emergency arrivals.

The simulation model described in [15] considers both bed and operating theatre resources. However, its scope is high-level, including multiple hospitals which draw from a centralised waiting list. At this level of detail beds are treated as a homogenous resource, therefore dependencies between wards within a single hospital cannot be modelled. [16] develops a generic framework for modelling hospital resources, and outlines a number of modelling considerations for OR/MS practitioners working in this domain. A model which incorporates the prescribed framework is developed and used to assess a set of competing theatre scheduling policies, and their downstream effect on bed occupancy. The model is also used to estimate the mean number of occupied beds per month using a stochastic representation of hospital processes, and shows that this can differ significantly from estimation methods which only make occupancy estimates based on averages. A similar approach has also been used in the simulation described by [17], in which a multi-ward hospital is modelled. However, this model appears to treat the elective admissions as a stream of exogenous demand, rather than a decision variable. [18] formulates a healthcare service as a set of connecting servers in their simulation model. The key decision is the resource allocation in each server. The simulation model is used to generate data to train a neural network model that will be used as a meta-model in their optimisation model. Although they apply the approach to a blood transfusion centre, their approach can be applied to a network of hospital wards.

Researchers have reported the development of comprehensive simulation models either at the hospital or unit levels. [19] proposes an interesting feature in their model which is the existence of a feedback loop between the state of the hospital and admissions decisions; allowing the admissions policy to dynamically respond to the state of the simulated hospital. [20] develops a whole-hospital simulation, designed at a level of genericity such that it could be parameterised and applied to most modern hospitals. The model has the ability to load a user-defined waiting list so that the admission of elective patients can be treated as a decision variable. In general, the whole-hospital simulation receives elective admissions from the waiting list component and emergency admissions from the accident and emergency component. These are used to generate output statistics which include time spent on waiting lists, elective cancellations and the number of patients who become outliers.

In summary, the potential benefits of using simulation for bed management are well established. However, their use is often limited to (tactical and strategic) planning decisions, and few have attempted to produce models which can help hospitals with short-term (operational) decision making. Even fewer have attempted to use data that are made available after the model has been developed (or even during run-time) to re-initialise or re-parameterise the simulation model. This is where Symbiotic simulation can help.

### Symbiotic simulation

The concept behind what we know today as symbiotic simulation is not new. Computer scientists use software-in-the-loop and hardware-in-the-loop simulation to test software and hardware prototypes, respectively. In this approach, the software or hardware to be tested is connected to a simulator that mimics the environment in which the software or hardware will be operating. This type of simulation is also called co-simulation. Similarly, other terms have also been used in different domains such as real-time simulation, online simulation, dynamic data-driven simulation, digital twins, etc. [21] was among the first to describe the architecture of the simulation in detail. The term symbiotic simulation itself was coined at the 2002 Dagstuhl seminar on Grand Challenges for Modelling and Simulation [22]. The initial definition was heavily influenced by dynamic data-driven application systems which put an emphasis on the ability of the simulation to control the physical system. [6] proposes a new definition that is less restricted. In the new definition, symbiotic simulation is “a close association between a simulation system and a physical system, which is beneficial to at least one of them.” In this paper, we use Aydt’s definition of symbiotic simulation.

[21] provides an overview of the requirements for symbiotic simulation (referred to as “online simulation”) and proposes the use of parallel models operating under alternative control policies, along with a single model operating under the current control policy. The performance of each model is analysed, and the physical system adopts the policy which generates the “best” simulated results (given the physical system’s current state) for the next planning period. Another important contribution of [21] is the discussion of “reactive” versus “proactive” decision-making using symbiotic simulation models (which is further formalised in [6]). In reactive mode, a symbiotic simulation is used to develop a plan at a point in time (a so-called “decision point”), possibly in response to a critical state in the physical system, which is implemented in the physical system until the next decision point occurs. The alternative is a “proactive” mode, in which the plan is updated between decision points as the physical system evolves. While either of these modes of operation could in theory be applied to operational bed management in hospitals, they are dependent on the rate at which the hospital’s databases can be synchronised with actual bed occupancy. For instance, if it is known that up-to-date data entry occurs only once per week, the hospital may be limited to reactive mode at weekly decision points.

[23] further develops the theory of symbiotic simulation by considering some of the challenges associated with their initialisation. Since symbiotic simulation models are initialised with a state reflecting the physical system and analysed via their transient behaviour, the accuracy of the initial conditions has a direct effect on the results. However, in systems where the state descriptors change quickly over time, the current state becomes a moving target. The authors describe two initialisation methods. The first involves maintaining a continuously synchronised parent model, from which any number of child models can be generated and run at any time. The second is more simplistic, and generates a model from a specially formatted file whenever a new simulation run is requested. Since the state of inpatient beds in our study evolve at a slower rate than the example applications described by [23] (traffic and pedestrian flow modelling), the initialisation method envisaged for this research bears more conceptual similarity with the second method. Additionally, hospitals may be able to choose times during the day when arrivals, discharges and transfers between wards are less likely to occur, thereby reducing the chance that the bed-state will change before the results are obtained.

From 2005, the symbiotic simulation literature has seen an increase in the number applications focusing on how existing technologies can be used to implement a symbiotic simulation. Key applications include manufacturing (e.g. [24–27]), unmanned aerial vehicles (e.g. [28–31]), transportation (e.g. [32]) and Data Centre operations (e.g. [33, 34]). A small number of applications of symbiotic simulation also exist in the context of health care, and it is clearly an area with scope for further research, development and application. Published applications predominantly focus on managing the operations of single departments, such as emergency departments (e.g. [7], [35–37]) and cardiac care (e.g. [8]). Our research attempts to further promote the application of symbiotic simulation in health care, in part by demonstrating how it can be used beyond the ED.

## A general hospital discrete-event simulation model

When the AGH approached us, they expressed interest in “… a core piece of work built around a predictive bed modelling tool for operational purposes …” After further email correspondence, we were supplied with an anonymised extract of the patient administrative (PA) database, for all patient episodes from the 1st of January 2010 to the 30th of June 2012. The PA data supplied by AGH is split into two databases, known as the *Care* and *Stay* databases. The Care database contains information about the type of treatment a patient is receiving; such as their specialty at any given time, and the identification code of the doctor responsible for their care. The Stay database contains information about the physical location of the patient; including the identification code of the bed they occupy and the ward on which they are staying. A new row in the database is created when any of these features change, and each row is populated with start and end dates/times. The data are sufficiently detailed for us to develop a simulation model.

### Key performance Indicator

**Fig. 1:**
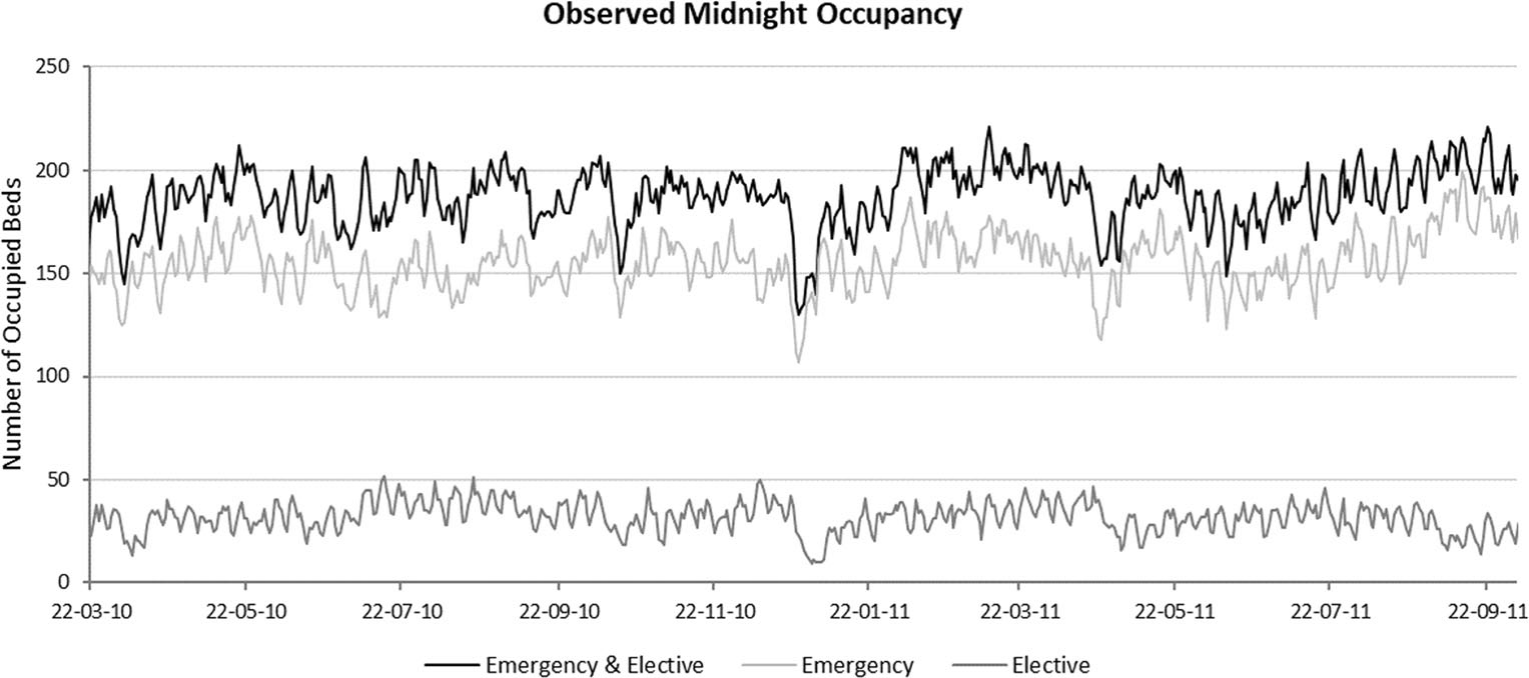
The emergency and elective midnight bed census during the observation period of 560 days

Since AGH is interested in a predictive bed modelling tool, the objective of the model is to estimate the number of occupied beds. The PA data allows us to obtain bed occupancy at any time of day. In this work we use a frequently-used metric called the midnight bed census, and its breakdown between emergency and elective patients, as shown in Fig. 1 (after excluding all patients who are admitted and discharged on the same day). Hence, we also use midnight occupancy level as the main metric in our simulation model and the simulation runs in discrete time, with each time unit representing one day of hospital operations.

### Conceptual model

**Fig. 2:**
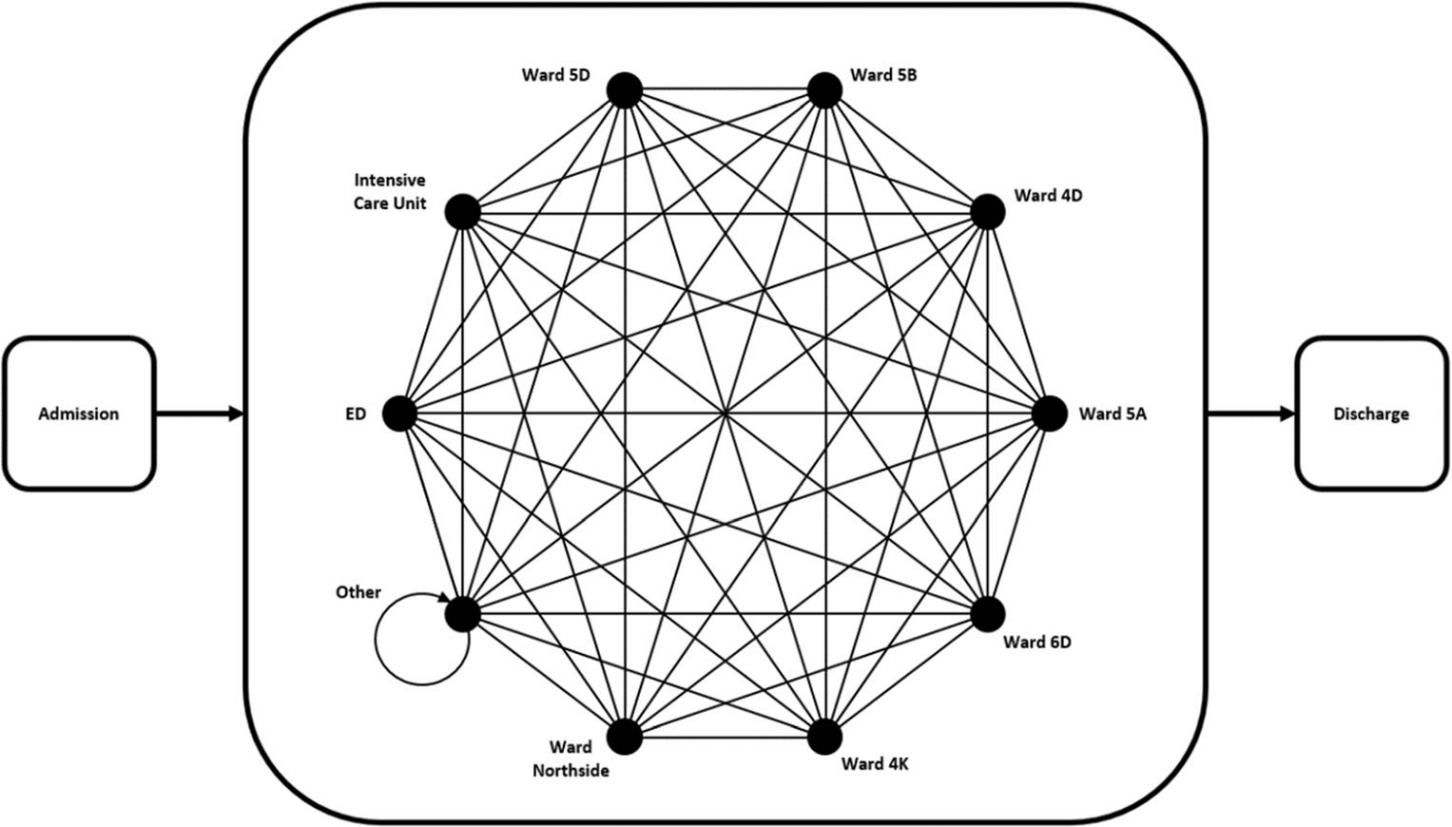
The network of ten individually modelled wards forms a complete graph

Since this project is concerned with estimates of inpatient bed occupancy at the ward level, the minimum level of structural detail includes a network of wards (see Fig. 2). Patient stays can be disaggregated into ward stay segments to parameterise each ward in the simulated network. However, modelling every ward which appears in the PA data is not considered sensible, as some wards rarely allow overnight stays, and hence have very little relevant data. On the other hand, omitting these wards would break the links in the ward network. A pragmatic approach is to model wards that make up 90% of the average occupancy individually, and aggregate information relating to the remaining wards into one pseudo-ward in the simulation (referred to as ward *Other*). This means that the population of interest is captured entirely, while modelling effort is reserved for wards which are individually significant.

**Fig. 3:**
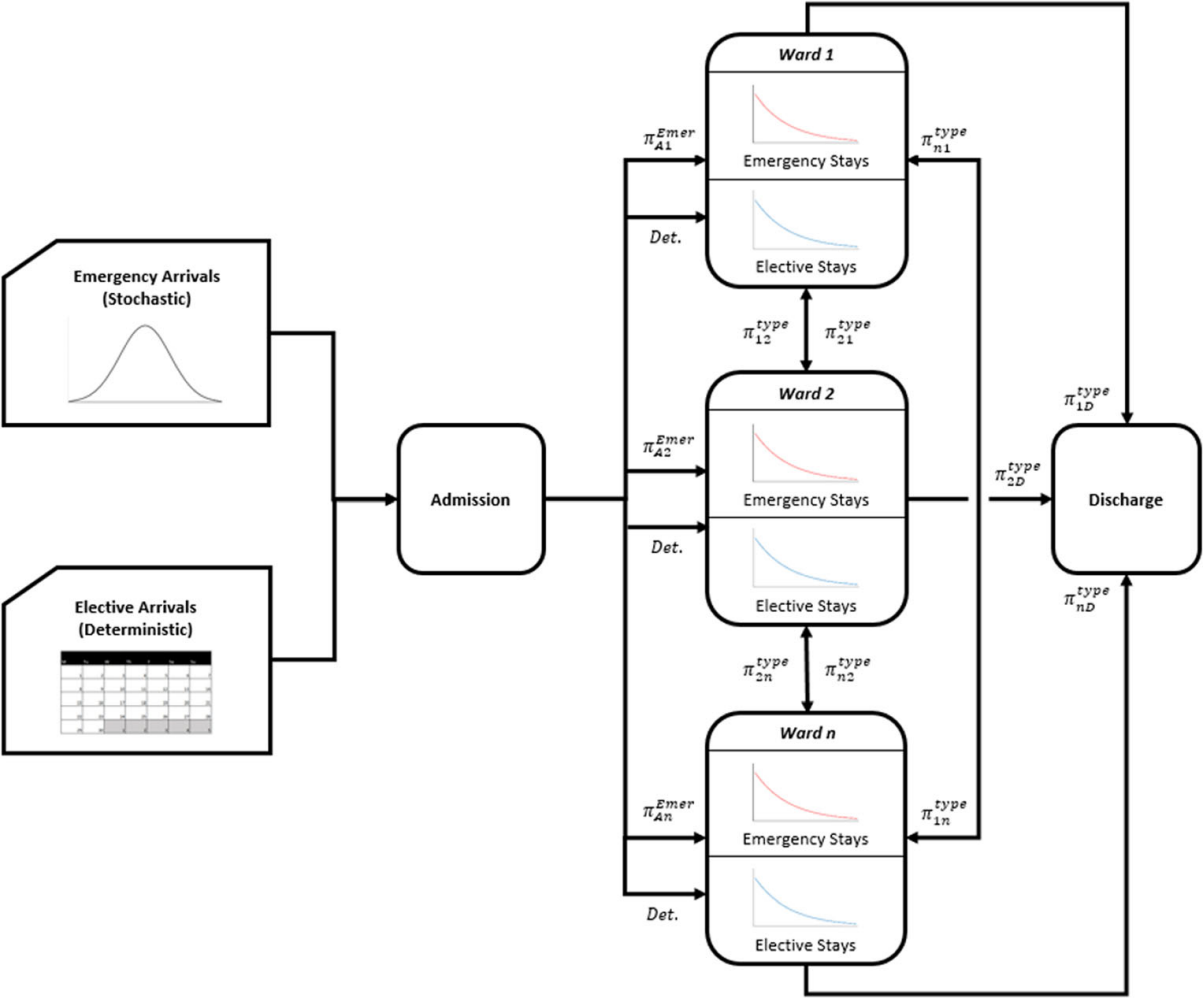
Schematic of the simulation model. The ${\pi}_{ij}^{type}$ represent the transition probabilities from ward *i* to *j* for each admission type (emergency/elective)

Figure 3 shows the structure of the model. The number of emergency admissions per day is modelled using an empirical distribution for each day of the week, in keeping with the random nature of emergency arrivals in a real hospital. The elective admissions are the decision variables in the model. Hence, elective admissions occur deterministically by choosing the day and ward of arrival for each elective patient in the planning horizon. If the probability of non-attendance can be estimated, this information can be used with the planned admissions pattern to emulate unexpected patient absence.

After being admitted to the hospital, patients occupy a bed for some period before being discharged or transferred to a bed on another ward. This period is known as the patient’s ward length-of-stay (WLOS). In the case of elective admissions, it may well be estimated by clinicians and planners responsible for scheduling procedures (which is the basis of one of the example applications presented in 6.2). However, there will still be random variation in length-of-stay from patient to patient. For emergency patients arriving at the hospital, length-of-stay may be even less predictable due to the unscheduled nature of their admission. For this reason, WLOS are modelled as random variables. Patients can stay in more than one ward while being treated in the hospital. Hence, the sum of their WLOS form their total length-of-stay (LOS) in the hospital. For readers who are familiar with the UK NHS, the terminologies are ward stay and spell, respectively.

Once a patient’s ward stay is over, they may be discharged from hospital or they can be transferred to another ward. If a transfer to another ward is necessary, the choice of ward is not only dependent on the patient’s clinical requirements, but also the availability of the resources needed to treat the patient, such as beds, nurses and monitoring equipment. The potential for uncertainty in the sequence of visited wards, along with the unknown types of emergency arrivals occurring in each planning horizon, justify the use of stochastic transfers between wards in the simulation. The probabilities which govern the transfers from each ward are estimated from the PA data by calculating the proportion of total departures moving to each subsequent ward or to discharge. Modelling transitions in this way is a simplification of the real transfer/discharge process, since the probabilities depend on the current ward and do not consider previously visited wards (memorylessness). However, this approach has been shown to work well in other models, see for example [20], and maintains the average patient flows seen in the real hospital.

### Infinite server assumption

In his review of the development of queueing theory and applications, [38] comments that despite their assumption of infinite resource, infinite server (i.e. uncapacitated) models can provide the basis for the analysis of offered load for multi-server systems with time-varying arrivals. Hospitals such as AGH can be viewed as a network of servers with time-varying patient arrivals. Whitt’s comment is confirmed by the literature in which various authors have used both analytical (e.g. [39–41]) and simulation (e.g. [8, 14, 42]) infinite server models to address the bed management problem in hospitals.

Infinite server models deliberately exclude real-world resource constraints, and hence produce simpler models. The use of simple models that are fit for purpose is a good practice for simulation modelling. In our case, infinite server models are well suited to estimate the probability of demand exceeding a certain level in the short term, which is a key piece of information for hospital managers trying to assess the risk of not being able to cope, given the number of beds at their disposal. Similar statistics could be derived using a fixed capacity approach, however doing so adds considerable complexity in terms of modelling the extent to which patients are turned-away.

As noted in section 1, our research objectives require a *proof of concept* symbiotic simulation model that can be used to investigate the issues of model development, model validation and model application. It is not the purpose of this paper to argue that the infinite-server model adopted here is the only, or ‘best’, way of modelling inpatient bed occupancies. There will certainly be occasions where it would be useful to increase the complexity of the model to reflect management decisions taken when wards are full, or for example to model the sorts of self-regulatory behaviours described in [43].

### Validation of DES model

**Fig. 4:**
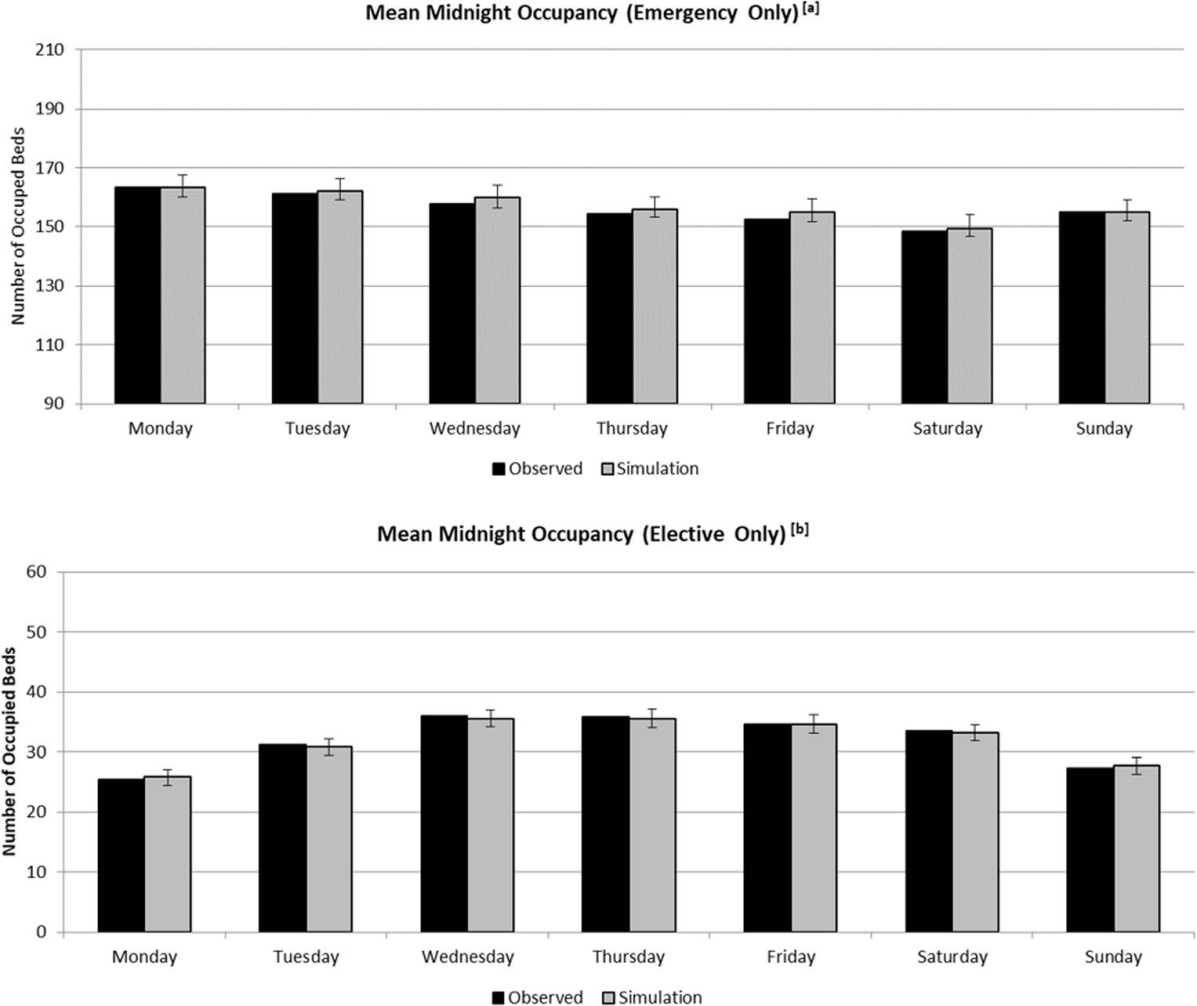
Mean midnight bed occupancy by weekday for emergency patients (a) and elective patients (b)

The conceptual model described in the previous sections is implemented in the Micro Saint Sharp simulation package. We applied white-box validation as defined in [44] whilst working with the AGH, and black box validation in which the model outputs were compared to some historic data generated by the physical system to confirm that the model displays similar performance characteristics when run under similar operating conditions. Figure 4 compares the observed mean midnight occupancy (by day of the week) with realisations of mean midnight occupancy derived from the simulation outputs for emergency and elective patients. The error bars within the bar charts are two-tailed 90% confidence intervals for the mean midnight occupancy derived from the simulation. The result shows that at the 10% significance level, there doesn’t appear to be any misspecification of the model in terms of mean occupancy on each day of the week at the admission type level (emergency/elective). Further validation tests of the DES could be carried out at this stage, however the main validation exercise needs to be carried out on the symbiotic simulation model.

## Symbiotic simulation development

This section describes the development of a symbiotic simulation model from the DES model described in Section 3. The objective is to demonstrate that in cases where we already have a validated (non-symbiotic) simulation model (as in some hospitals), we may not need to develop the symbiotic version from scratch. This section shows that two key functionalities can be added to the existing simulation model to make it symbiotic. The functionalities are the ability to load the state of the physical system at run-time, and conditional service-time distributions which govern patients’ lengths of stay.

### Ability to load the state of the physical system at run-time

This functionality ensures that the simulation can be initialised to the state of the physical system being modelled to investigate how the physical system might evolve given its current state. In general, having a communication line with the physical system means that the physical system state can be queried by the simulation model at any time. However, in a hospital setting this may not be possible, since data entry into the patient administration system may not occur automatically. This may change in the future as more data are being collected automatically in hospitals via sensors, RFID, mobile electronic devices and other technologies. Likewise, future commercial simulation software is likely to include the functionality to interface with these real-time data sources. Currently, most commercial simulation tools (including Micro Saint Sharp) have the ability to read a file that can be used to initialise a simulation. Hence, a symbiotic simulation can be implemented using existing software packages that share this feature.

The system state that is needed to make the simulation model in Section 3 symbiotic consists of:

- The number of emergency admissions resident on each ward.
- The number of elective admissions resident on each ward.
- The day of the week on which each patient was admitted.
- The amount of time already spent on the current ward for each patient at the time the state data is collected.

The first and second pieces of information are the most obvious requirements when attempting to describe the state of the model. The third piece of information relates to the way in which ward length-of-stay is modelled. More specifically, a statistically significant relationship was found between the day of the week on which a patient was admitted, and the length of time they subsequently spent in hospital. Thus, day of admission information is required for each patient’s ward length of stay to be drawn from the appropriate empirical distribution. The use of empirical distributions aligns closely with the data-driven nature of symbiotic simulation. However, this does not prevent the use of theoretical distribution when appropriate. The fourth piece of information ensures that the patients who are resident on a ward when the state data is captured are loaded as simulation entities who have spent the same amount of time on the ward.

### Conditional distributions

In systems where each “job” has a service time, new jobs have service times sampled from their underlying probability distributions, whereas any job currently in service when the simulation is initialised should be loaded into the model with its remaining service time. In our case, ward length-of-stay (WLOS) is the service time of interest and is treated as a random variable conditional on admission type, weekday of admission and hospital ward. Therefore *remaining* length-of-stay of a patient already in a ward when the simulation is initialised is stochastic, and cannot be known at run-time. However, this remaining WLOS is likely to be dependent on the time already spent on the ward and hence necessitates the use of *conditional* distributions (also used in the analytical models of [45–47]).

The conditional WLOS distribution for each resident patient is straightforward to derive, given their time already spent on the ward, and the marginal distribution of length of stay (accounting for admission type, weekday of admission and hospital ward) applicable to the patient. Suppose the random variable *T* represents the total number of nights a given patient will spend on the ward, and that when the simulation is initialised the patient has already been on the ward for *s* midnights. The random variable *R* = *T* − *s* therefore represents the number of midnights the patient remains on the ward after the simulation is initialised. If the CDF of total length of stay, *F*_*T*_(*t*), is estimated using the empirical data, the conditional CDF *F*_*T*_(*t*, *s*) = *ℙ*{*T* ≤ *t*| *T* ≥ *s*} can be obtained simply using the formula:

1 $$ {F}_T\left(t,s\right)=\mathbb{P}\left\{T\le t|T\ge s\right\}=\frac{F_T(t)-{F}_T\left(s-1\right)}{1-{F}_T\left(s-1\right)} $$

Since *R* is the difference between *T* and *s*, the sampling distribution for *R* is then readily given by:

2 $$ {F}_R\left(r,s\right)=\frac{F_T\left(s+r\right)-{F}_T\left(s-1\right)}{1-{F}_T\left(s-1\right)} $$

For a given *s*, realisations of *R* can then be drawn from *F*_*R*_(*r*, *s*) using the inverse transform sampling method, and these realisations represent remaining length of stay on the ward, given length of stay already spent on the ward at the time the simulation is initialised. Most commercial simulation software will allow the user to specify any function for the sampling distribution of activity durations, and hence this is simple to implement in such software.

It is worth noting that conditional WLOS does not need to be considered for models whose service times are best described using exponential distributions (when service times are continuous) or geometric distributions (when service times are discrete), due to the memoryless property. However, memorylessness does not apply to the empirical distributions from which WLOS is drawn in this model.

## Validation for stochastic symbiotic simulation using Δ-method

**Fig. 5:**
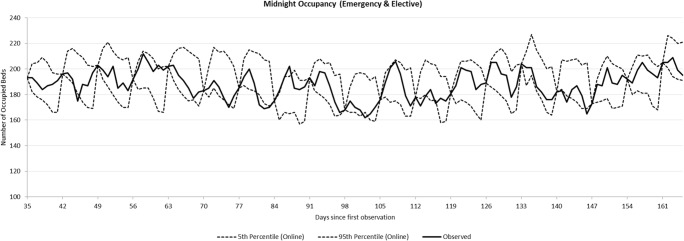
90% prediction intervals of bed occupancy generated by the symbiotic simulation for all wards combined, for all admissions

One of the characteristics of stochastic symbiotic simulation (e.g. built from Discrete-Event Simulation or Agent-Based Simulation) is that the simulation needs to be re-initialised with the data from the physical system it is meant to control. Hence the distribution of a metric estimated by the stochastic symbiotic simulation (e.g. midnight occupancy in this paper) changes as a function of the elapsed time from initialisation. Figure 5 shows the result from running our symbiotic simulation for 100 replications, re-initialising it on the Monday of each week over an 18-week period. The choices of Monday and weekly updates are arbitrary, and are intended to provide a realistic example. Note that the bed occupancy status each Monday is unique, and has a unique trajectory over the following 6 days which we wish to compare with our simulated results. Our validation analysis below is based on 80 weeks of data, however we limit ourselves to 18 weeks in Fig. 5 so that the detailed nature of the results is clearly visible.

Figure 5 shows a typical example of a symbiotic simulation output. The thick line shows the observed metric (in our case, midnight occupancy). The dashed lines show the 5th and 95th percentiles (90% prediction intervals) of the midnight occupancies from the symbiotic simulation. First, this figure shows that the prediction intervals cover the observed data well (87% of observations fall within their corresponding 90% prediction interval). Secondly, we can see that the prediction intervals collapse to the observed midnight occupancy every Monday where the simulation is re-initialised. This is the primary feature which distinguishes symbiotic simulation from non-symbiotic simulation. Based on this feature, we propose the ‘Δ-Method’ as a validation test for stochastic symbiotic simulation which compares the distribution of the simulation outputs over time, to that of the historic data.

The validation test is based on comparing the distributions of simulated and observed changes in ward occupancies over the range of prediction periods of interest *h* (e.g. 1 to 6 days ahead). With 560 days of observed data, for each ward we have 560-*h* observations of:

3 $$ {\Delta }_{t,h}={M}_t-{M}_{t+h} $$

where *M*_*t*_ represents the measure of metric *M, t* days from the start of the observation period.

If the symbiotic simulation is initialised at time *t*_0_, then ${M}_{t_0}$ will take the same value in both the simulation and the physical system. In our case the length of the planning horizon is assumed to be one week and the symbiotic simulation model is initialised weekly, hence there are 80 weekly initiation points, and 6 empirical distributions of Δ_*h*_ (one for each day of the planning horizon (Δ_1_, … , Δ_6_)). If *F*_*simulation*_(*δ*_*h*_ ) and *F*_*physical system*_(*δ*_*h*_ ) denote these empirical cumulative distribution functions over the support of *∆*_*h*_, denoted by *δ*_*h*_, then the coordinates (*F*_*simulation*_(*δ*_*h*_ ), *F*_*physical system*_(*δ*_*h*_)) form a *probability-probability plot* or *P-P plot*, see for example [48] for a description of their use as a graphical technique. If the distributions are similar, the coordinates will lie close to the identity line (*y* = *x*), providing a visual indication of the similarity of the distributions of Δ_*h*_ at each possible elapsed time *h* from initialisation, or equivalently, on each of the *h* days in the planning horizon.

To illustrate how Δ-Method works, we apply it to our symbiotic simulation. In this case we are interested in the midnight occupancy of each ward. Hence, our Δ_*h*_*-occupancy* on ward *w* is:

4 $$ {\Delta}_{t,h}^w={M}_t^w-{M}_{t+h}^w $$

where ${M}_t^w$ represents midnight occupancy *t* days from the start of the observation period on ward *w*.

**Fig. 6:**
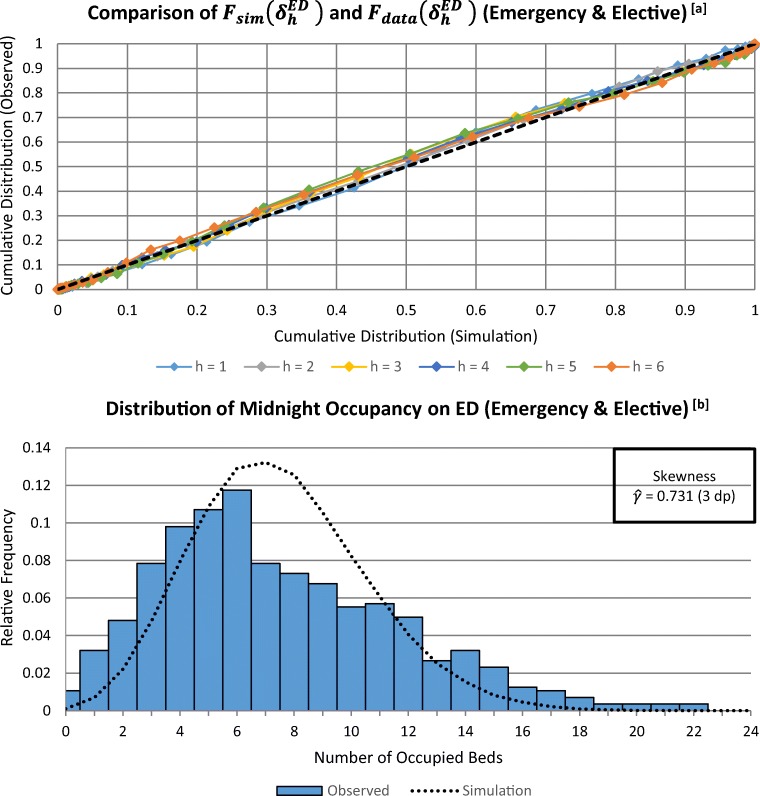
**a** The P-P Plot of **Δ**_***h***_-occupancy at each time from initialisation (***h***). **b** Histogram of midnight occupancies recorded on the ED during the 560-day observation period, overlaid with the estimated p.m.f generated by the simulation (ignoring time-dependence)

**Fig. 7:**
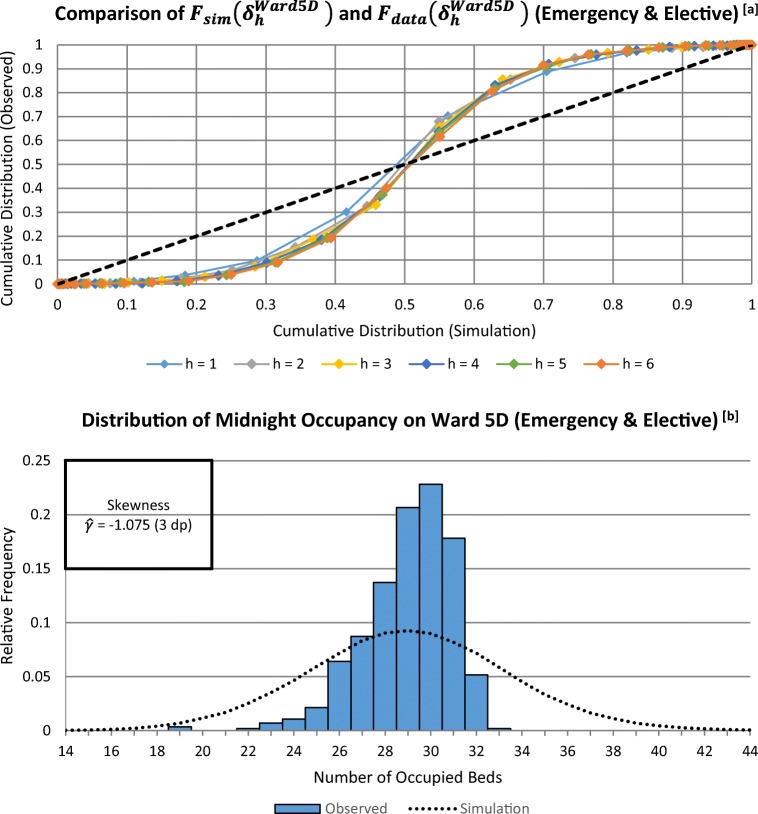
**a** The P-P Plot of Δ_*h*_-occupancy at each time from initialisation (*h*). **b** Histogram of midnight occupancies recorded on Ward 5D during the 560-day observation period, overlaid with the estimated p.m.f. generated by the simulation (ignoring time-dependence)

Figures 6a and 7a show the results of applying the ‘Δ-Method’ for two different wards, the ED ward and ward 5D. Each figure compares the six cumulative distributions of Δ_*h*_-occupancy observed in the historic data, with equivalent distributions generated by the output of 100 replications of the symbiotic simulation model, for the ED ward. The results in Fig. 6a are typical of many wards across the hospital, showing good agreement when compared to the empirical distributions of Δ_*h*_-occupancy across the six-day planning horizon. This indicates that the simulation model, including the infinite server assumption, seems to be representing the performance of these wards quite well. However the result in Fig. 7a indicates problems with using the model if its purpose were to predict occupancies for ward 5D. Here the P-P plots show that the distributions from the observed data have less cumulative probability than the simulated distributions below their respective medians (for the same values of the support), however this difference reduces towards the point (0.5,0.5), and changes to a positive difference above it. With both the simulated and observed data having very similar medians for each value of *h*, this pattern is indicative of lower variance in the distributions plotted on the vertical axis, compared to the distributions plotted on the horizontal axis.

Figures 6b and 7b show the results of more traditional validation tests, simply comparing the overall (i.e. averaged over all 80 weeks) observed and simulated occupancy distributions for the ED and for Ward 5D. Whilst obtaining these overall distributions are not the purpose of the symbiotic simulations, they do help understand the strengths and weaknesses of the chosen model. For the ED the distribution of real midnight occupancy is *positively-skewed*, is quite unlikely to be near, or at its maximum capacity, and hence is relatively well represented by infinite-server assumption. On the other hand, as Fig. 7b shows, the capacity of Ward 5D is often reached, which stops further patient stays from occurring. This behaviour will clearly not be mimicked when assuming infinite capacity wards. However, as noted in Section 3.3, one important use of infinite-server models is to warn management of the likelihood of demand exceeding capacity, rather than attempting to model the detail of the possible consequences. Hence, as will be seen in Section 6, whilst the validation test for Ward 5D warns us that it will not fully reproduce actual ward occupancies, it can nevertheless provide a valuable warning that there will be occupancy problems to be dealt with.

In summary, this section has focused on the development of a new validation technique suitable for stochastic symbiotic simulation in which the time-dependence of the simulation outputs is accounted for, and which are well suited to bed management applications. By defining the Δ_*h*_ random variable, the observed metric can be pooled in such a way that comparisons can be made with the simulated metric, whose distribution evolves with time-from-initialisation. Since Δ_*h*_ is analysed via a comparison of the entire empirical distribution function, differences in trend, variability, or cycling behaviour which may occur over time, can all be detected.

## Example applications

We present the following two examples to demonstrate how the symbiotic simulation could be used in practice.

### Early warning system

**Fig. 8:**
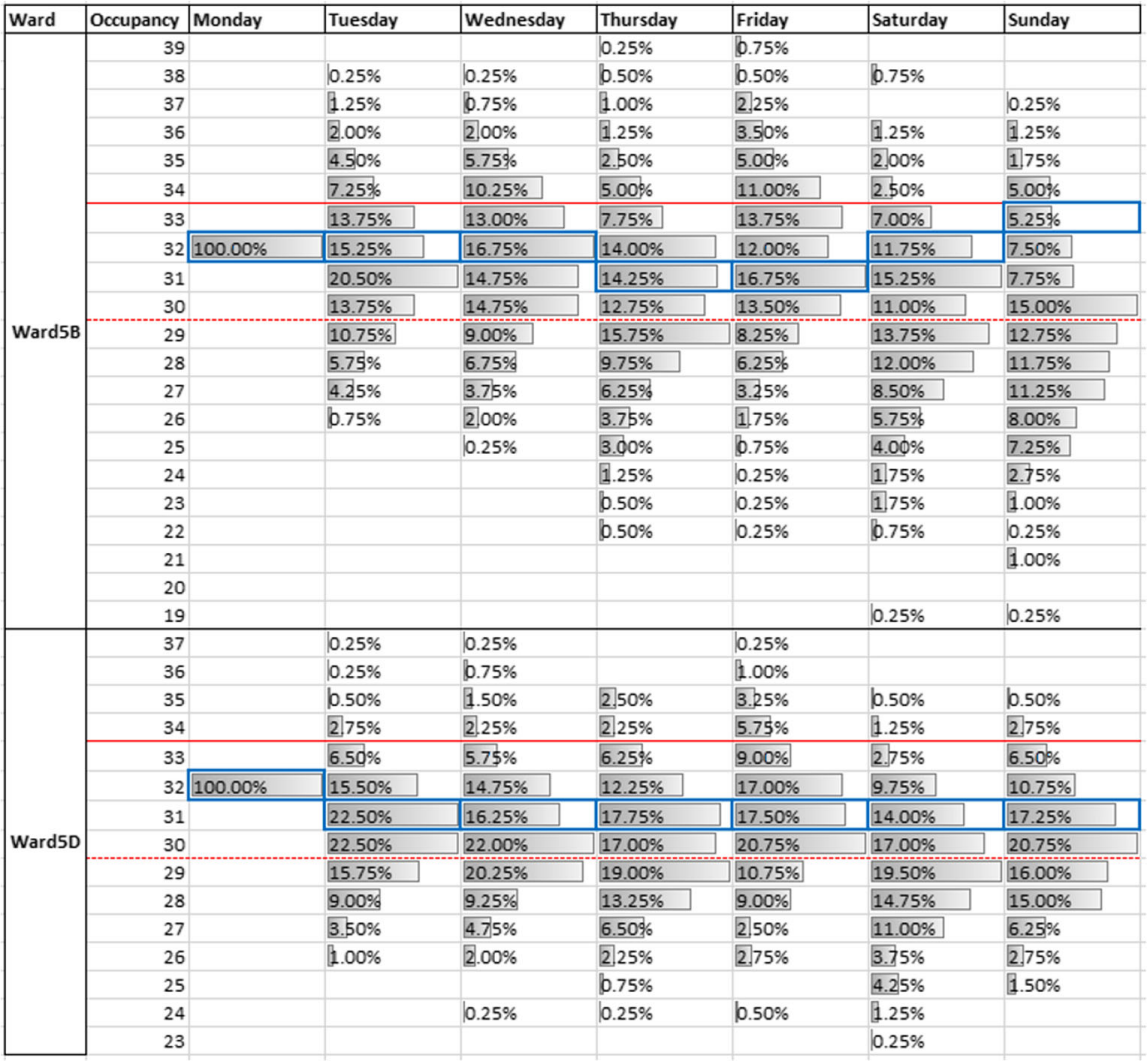
Distributions of midnight occupancy generated by the symbiotic simulation on Ward 5B and 5D

Motivated in part by the needs of AGH, the first example application demonstrates how the symbiotic simulation model could be used as an early-warning system to anticipate days in the planning horizon when the demand for beds is at risk of exceeding the maximum capacity of the wards. A particularly busy week is chosen from the PA database, and the symbiotic simulation is used to assess the likelihood of demand exceeding capacity for the observed elective schedule. We initialise the symbiotic simulation with the PA data on Monday of the chosen busy week and run the symbiotic simulation for 400 replications. Based on our testing, 400 replications resulted in sufficiently stable midnight occupancy distributions when only the random seed was changed. For brevity, only Wards 5B and 5D are shown in Fig. 8 since their real midnight occupancies sit above their respective 90% capacity thresholds for every day during the week; making them suitable for demonstrating the simulation’s use as an early warning system.

Figure 8 shows the histograms for each day during the busy week for two of the modelled wards. The dashed red line represents the 90% occupancy threshold, while the solid red line represents the maximum occupancy of the ward. The solid blue cells indicate the actual level of midnight occupancy which the ward experienced. For both wards, the distributions derived from the simulation outputs indicate that midnight occupancy is more likely to be above the 90% occupancy threshold, rather than below. Therefore, the symbiotic simulation could have been used to warn hospital staff of the high probability of high midnight occupancy for most days during the week.

In addition to indicating the days when midnight occupancy is likely to be above the 90% threshold, Fig. 8 also shows that the symbiotic simulation can be used to anticipate days where the demand for beds might be more than the number of available beds. This shows how our simple uncapacitated model can be used as an early warning system by showing the probability of over-occupancy. When the probability of exceeding capacity is high, as in this example, the hospital manager has more time to plan for some sort of preventative action, such as an alternative elective schedule.

Suppose we now want to evaluate two possible revisions to the elective admissions schedule for the busy week in question. Many of the admissions take place on the Other ward which is an aggregate of smaller wards, meaning the actual ward of admission has low average midnight occupancy. The actual admissions schedule for the busy week is shown in Table 1, and the proposed revisions are highlighted. The postponement schedule postpones one patient from Tuesday to Wednesday and two patients from Thursday to Friday. The cancellation schedule is the same as the postponement schedule but also cancels one patient from Thursday.

**Table 1:**
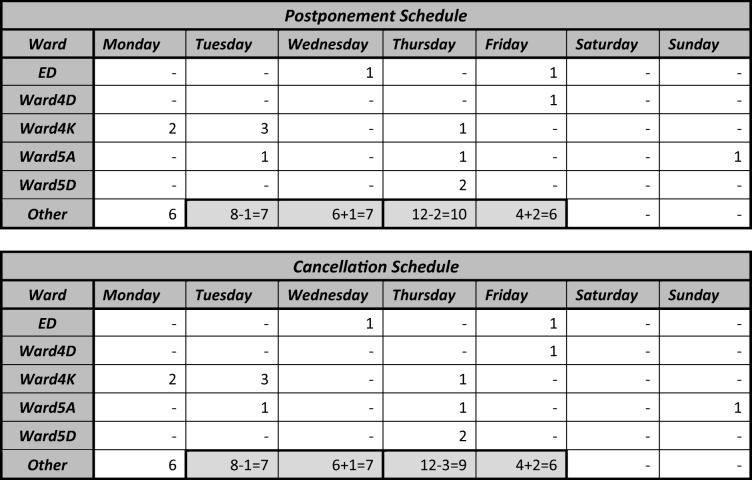
Alternative elective schedules: postponement (top) and cancellation (bottom)

**Fig. 9:**
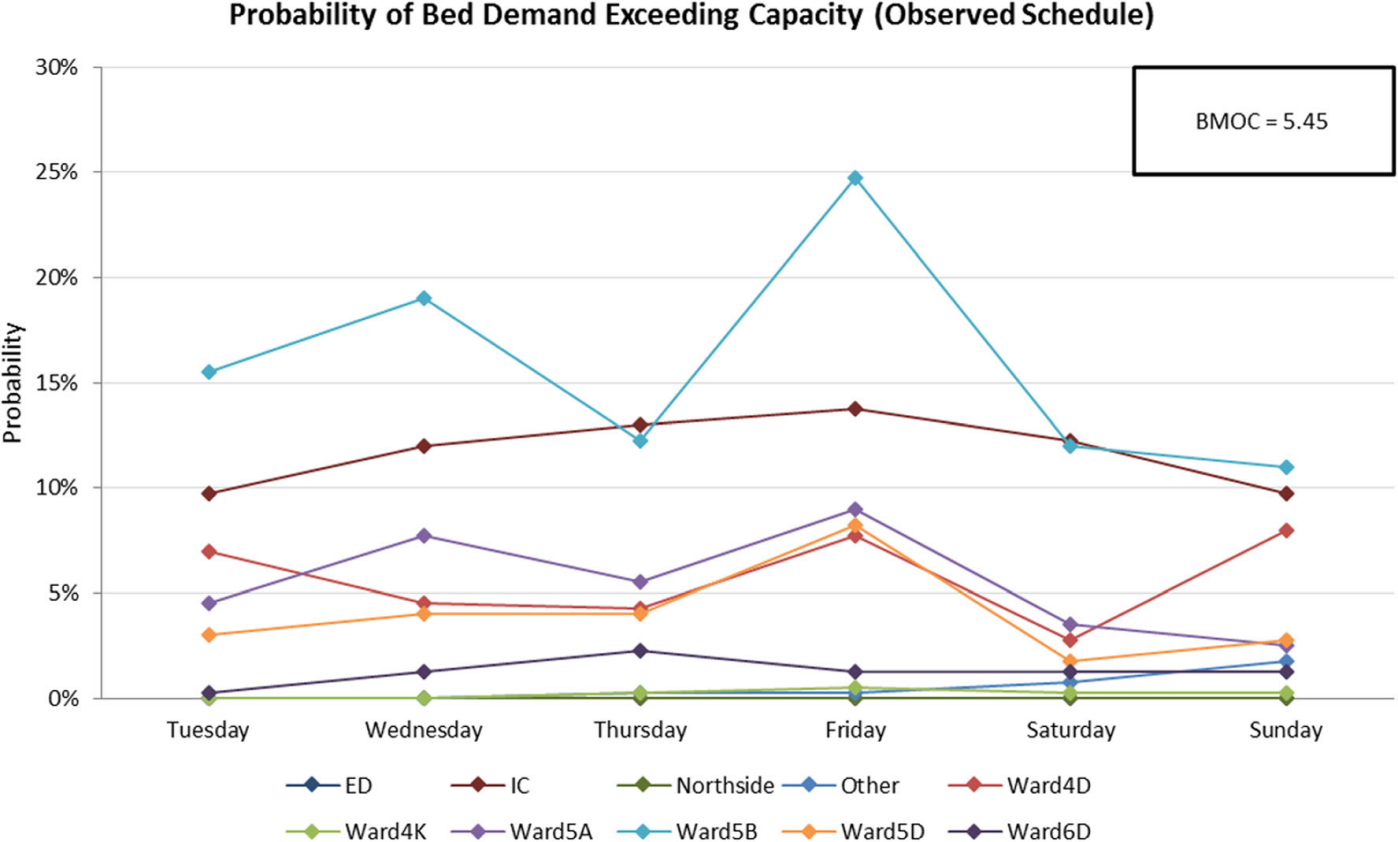
Symbiotic simulation estimates of the probability of the demand for beds exceeding total capacity on each of the ten modelled wards during a busy week using the observed schedule

**Fig. 10:**
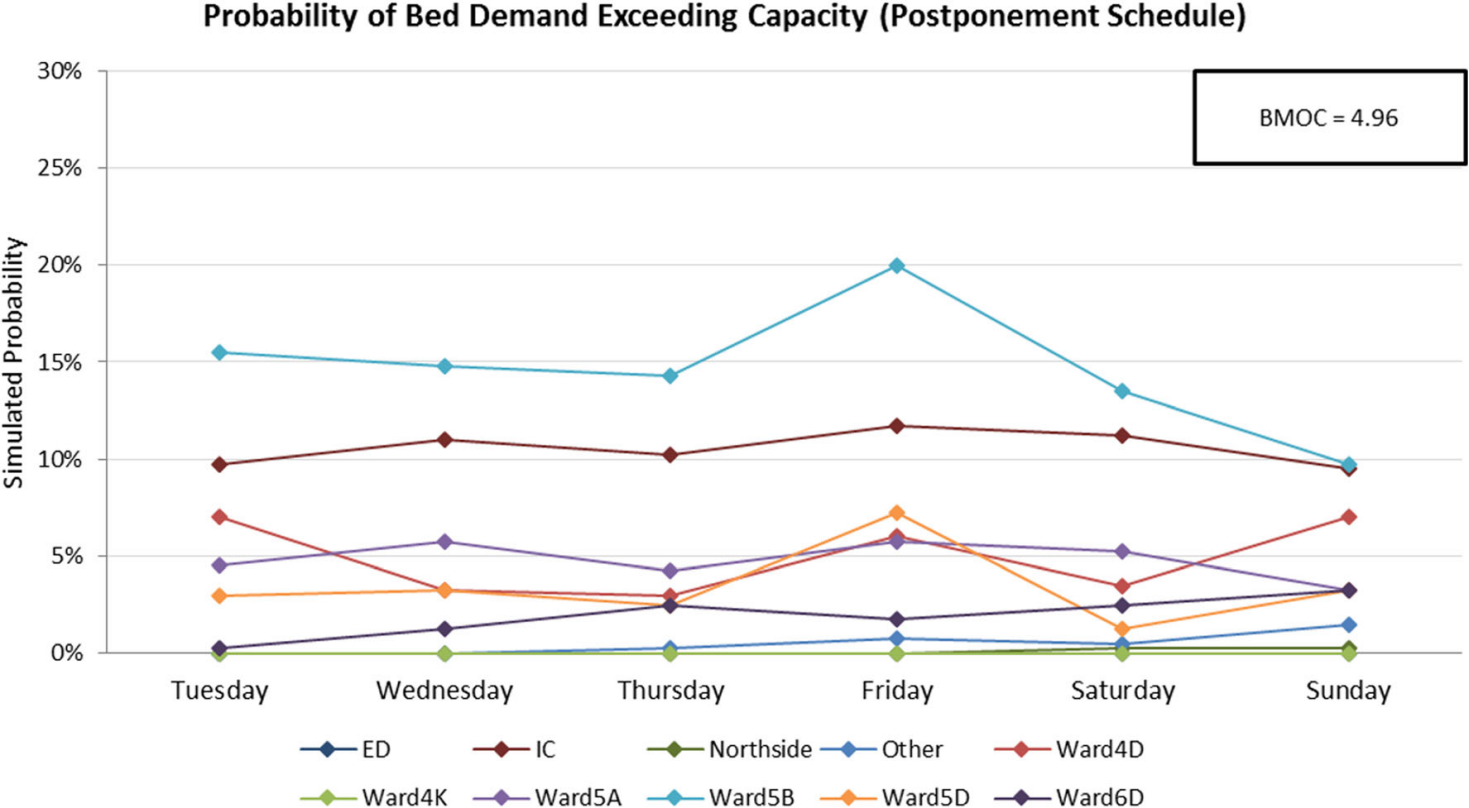
Symbiotic simulation estimates of the probability of the demand for beds exceeding total capacity on each of the ten modelled wards during a busy week using the postponement schedule

**Fig. 11:**
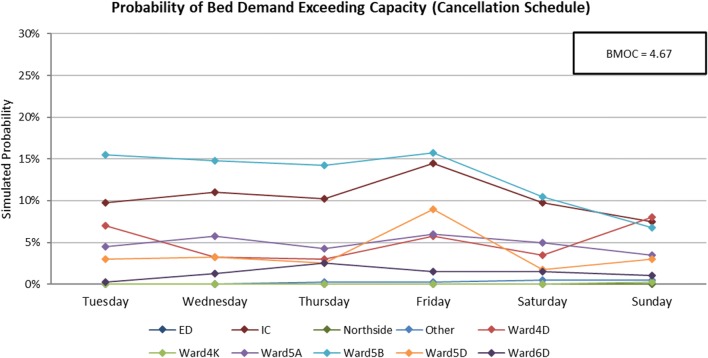
Symbiotic simulation estimates of the probability of the demand for beds exceeding total capacity on each of the ten modelled wards during a busy week using the cancellation schedule

Clearly, the likelihood of demand exceeding capacity should be assessed for every ward, in a holistic way. Hence, to compare the three elective schedules (observed, postponement and cancellation), we chart the probability of bed demand exceeding the maximum capacity on every simulated ward as shown in Figs. 9, 10 and 11. Monday is excluded because this is when the symbiotic simulation is initialised. Added to the figures are estimates of ‘bed-midnights over capacity’ (BMOC), which is the total number of beds in excess of each ward’s capacity, for all wards, summed over the midnights in the planning horizon (based on [49]). A single estimate is obtained by averaging this metric over all replications for the simulated week.

The charts clearly show that Ward 5B is the most likely ward to encounter capacity issues for most days of the week if the observed schedule is not revised. The Postponement Schedule is able to reduce the peaks in probability on both Wednesday and Friday for Ward 5B, and as expected, increases the probabilities on Thursday and Saturday. However, the level of risk is now more even across the week, and hospital managers may consider these two days to be in a better position to accommodate additional patients than Wednesday and Friday. The estimated BMOC also sees a decrease of 0.49 bed-midnights, also indicating a net improvement across all wards using this schedule. The Cancellation Schedule further reduces the probability of Ward 5B encountering capacity issues by approximately 5%, and BMOC by 0.29. Whilst operation cancellations are clearly undesirable, hospital managers may nevertheless sometimes need to trade-off this sort of an improvement against the consequences of cancelling the patient.

This example illustrates how our symbiotic simulation might be used to best reduce the likelihood of excessive bed demand, thereby balancing emergency and elective workloads.

### The benefit of additional information

One of the benefits of symbiotic simulation is the ability to use newly available data that are made available after the simulation model is developed; including at simulation run-time. This example investigates the potential for improving the accuracy of results generated by the symbiotic simulation (e.g. estimates of bed demand) by making use of additional (and potentially subjective) patient information that might be available at simulation run-time. This could include information about the location of subsequent ward stays, or the likelihood of requiring an Intensive Care bed. In this example, the additional information being considered concerns the length-of-stay of patients on the elective admissions schedule, and the remaining length-of-stay of any patient (emergency or elective) who occupies a bed when the symbiotic simulation is initialised. This information is used as a proxy for the Estimated Date of Discharge (EDD) which [50, 51] emphasises is an essential care coordination tool within the UK.

EDD (and thus, estimated length-of-stay) aligns with the symbiotic simulation method particularly well. New system state data is already read into the model at regular intervals, and this can easily be augmented with information about a patient’s condition (i.e. how long they are expected to stay) as it develops. For the incoming elective patients, clinicians will have an approximate EDD in mind to help manage hospital resources, and to inform patients of the time they can expect to spend in hospital. For the emergency patients already in the hospital, clinicians should have more information than when they were first admitted, which can contribute to an EDD. In fact, [49] recommends that an EDD should be set at the first consultant review, and set no later than the first consultant ward round the following morning. Therefore, estimates of remaining length-of-stay should also be available for most, if not all acute patients occupying a bed.

However, clinician’s assignment of an EDD is by no means a guarantee that the corresponding patient will be discharged on their estimated date. Factors such as variation in individual recovery times, and complications associated with treatment, can contribute to differences between the EDD and the actual date of discharge. Therefore, as part of assessing the value of using discharge date estimates in a symbiotic simulation, it is also important to consider how accurate they might be.

Although estimates of LOS/EDD were not explicitly provided by the AGH participating in this study, the actual ward lengths-of-stay can be loaded from the PA data, retrospectively. The use of actual observations would represent a scenario in which clinicians were able to perfectly predict LOS. To model the uncertainty associated with clinicians’ LOS estimates, we considered information about LOS on each patient’s current ward, and introduce a parameter *d*, which controls the proportion of correct estimates of current ward LOS. Hence, in the simulation, clinicians make correct estimates of LOS with the probability of *d*, and inaccurate but still good estimates (by sampling from the empirical LOS distributions) with the probability of 1-*d*. The symbiotic simulation is run for 400 replications using the same settings used in Section 5.

**Fig. 12:**
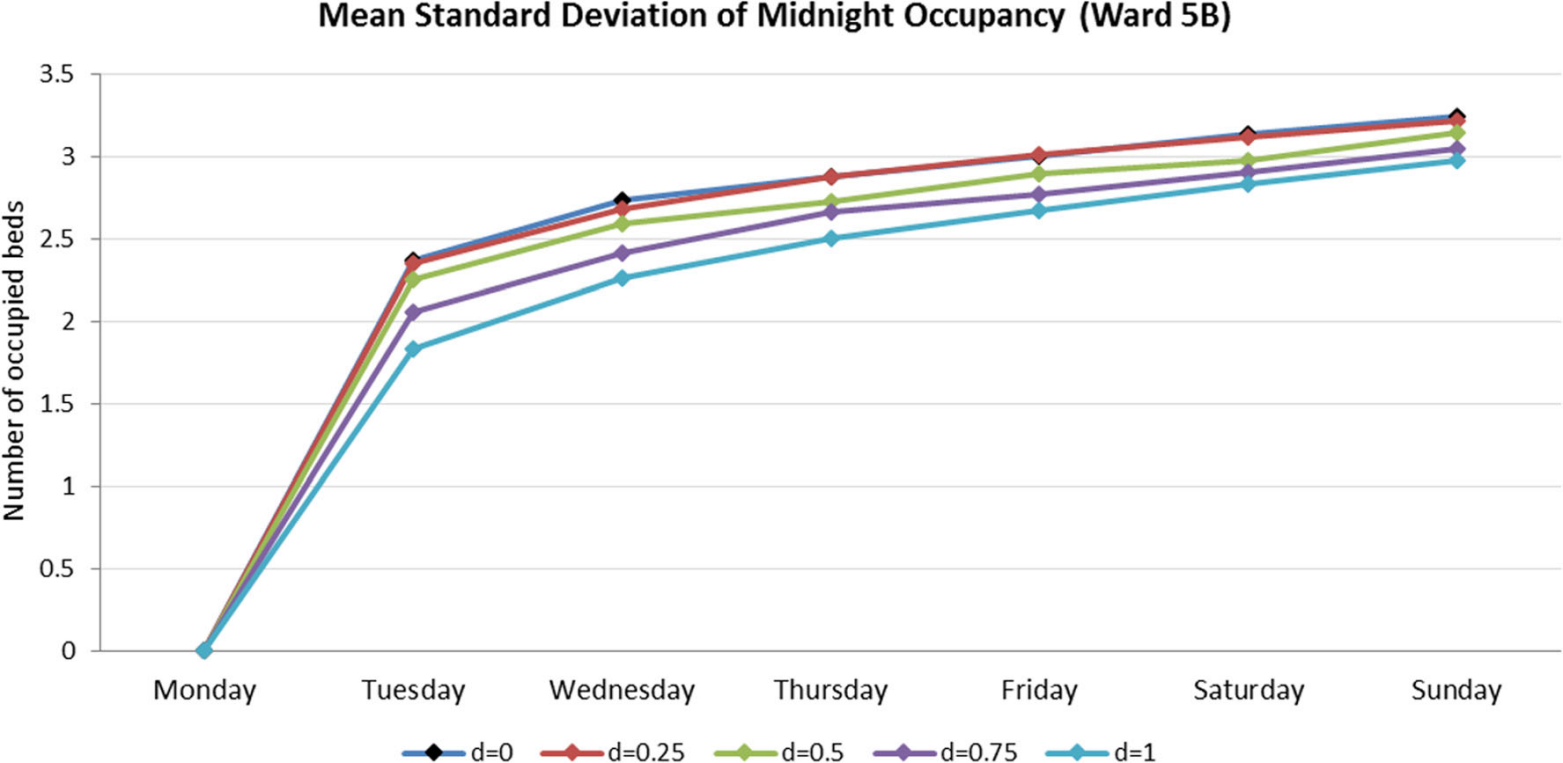
The estimated standard deviation of simulated midnight occupancy for each day in the observation period is averaged over the day of the week on which it occurs

Figure 12 shows the impact of different LOS error levels (*d*) on the standard deviation of the midnight occupancy on Ward 5B, where *d* = 0 means no additional information is used (as in Section 5) and *d* = 1 means the additional information is accurate for all patients. Ward 5B has been chosen as an example (other wards with similar characteristics display similar features).

As one would expect, as *d* increases the standard deviation of midnight occupancy on Ward 5B decreases, resulting in increased accuracy of the midnight occupancy estimates. However, Fig. 12 also suggests that clinicians’ EDD accuracy should be greater than 25% for those improvements to be discernible from the existing model in which the additional information is not used. Used in this way, the case can be made for the collection and inclusion of potentially subjective data, by also simulating the levels of accuracy at which they become useful. Such improvements could enable hospital managers to make better decisions, especially when the hospital is busy, as shown in the earlier examples.

## Conclusion and future work

The main contributions of this paper are concerned with model development, model validation and model application for symbiotic simulation models in the context of operational management of inpatient beds. In particular we have shown how a symbiotic simulation can be developed from an existing and validated (non-symbiotic) simulation model. This should reduce the cost of implementing and validating the symbiotic simulation model and promote model reuse.

The second contribution is the development of the Δ-Method; whilst based on the simple principle of compared simulated and observed outputs this new validation technique is suitable for validating a stochastic symbiotic simulation models and is well-suited to bed management problems. While other methods for validating symbiotic simulations are known to exist (such as [7]), the Δ-Method is the first known technique to consider the full distribution of the simulation outputs over time, rather than using select summary statistics. Furthermore, whilst developed for our healthcare context, it also provides a new method to aid the validation of symbiotic simulation models more generally.

The third contribution is in the form of example applications which show how our symbiotic simulation can be used in practice and something of their potential value.

Whilst these are the main contributions of the work, we also note that the data requirements of the symbiotic model were quite manageable, and were met by extracts from AGH’s patient administrative (PA) database. Clearly, in practice, the outcome of the model validation might have been to request a more complex model, in which case the data required for calibration could become considerably more demanding.

Reflecting on Aydt’s definition of symbiotic simulation [6] as “a close association between a simulation system and a physical system, which is beneficial to at least one of them”, we note that both example applications in section 6 show the potential value of this approach. However we also note that they also warn the potential user that the value of recent information on the physical system can wear off quite quickly as the desired prediction period for the application increases.

In the future, we plan to extend our work to include two further components into our symbiotic simulation model. The first component provides an optimisation model to find a good elective patient schedule given the availability of the new information when the simulation is re-initialised. The second component provides a learning algorithm so that the symbiotic simulation can learn by adjusting its parameters to improve the accuracy of the outputs. In a separate stream, we plan to apply the proposed development approach and validation method to different problems.
